# Perverse schobers and Orlov equivalences

**DOI:** 10.1007/s40879-023-00628-x

**Published:** 2023-04-28

**Authors:** Naoki Koseki, Genki Ouchi

**Affiliations:** 1 The University of Liverpool, Mathematical Sciences Building, Liverpool, L69 7ZL UK; 2 Graduate School of Mathematics, Nagoya University, Furocho, Chikusaku, Nagoya, 464-8602 Japan

**Keywords:** Perverse schobers, Calabi–Yau hypersurfaces, Mirror symmetry, Derived factorization categories, 14F08, 14J33, 32S60

## Abstract

A perverse schober is a categorification of a perverse sheaf proposed by Kapranov–Schechtman. In this paper, we construct examples of perverse schobers on the Riemann sphere, which categorify the intersection complexes of natural local systems arising from the mirror symmetry for Calabi–Yau hypersurfaces. The Orlov equivalence plays a key role for the construction.

## Introduction

### Motivation and results

A *perverse schober* is a conjectural categorification of a perverse sheaf introduced in a seminal paper [24] by Kapranov–Schechtman. There is a well-known way to define the notion of local systems of categories, which are the simplest examples of perverse schobers. However, it is not clear how to define perverse sheaves of categories in general. At this moment, a general definition is only available on Riemann surfaces and on affine spaces stratified by hyperplane arrangements [15, 24]. A key observation for the categorification in the case of Riemann surfaces is the classical result of [8, 16, 17] that describes the category of perverse sheaves on a disk in terms of quiver representations. Quiver representations consist of linear algebraic data, and hence we can categorify them by replacing vector spaces and linear maps with categories and functors, respectively. More precisely, we will use the notion of *spherical functors* between dg categories developed in [1]. A lot of interesting classes of perverse schobers have been constructed via birational geometry [9, 12, 13, 34, 35] and symplectic geometry [14, 24, 25].

In this paper, we construct new examples of perverse schobers on the Riemann sphere $\mathbb {P}^1$, which arise from mirror symmetry of Calabi–Yau hypersurfaces. Let $X \subset \mathbb {P}^{\,n+1}$ be a smooth hypersurface of degree $n+2$, which is a Calabi–Yau variety of dimension *n*. Then there exists the mirror family of *X* over , with the unique orbifold point at $0 \in \mathbb {P}^1$. By a variation of complex structures, we obtain a natural homomorphismwhere $X^\vee$ denotes the mirror of *X*. Applying the conjectural mirror symmetry equivalence $D^\pi \textrm{Fuk}(X^\vee ) \simeq D^\textrm{b}(X)$ (partly proved in [30–32]), we get a homomorphism1.1which we think of as a local system of categories with the fiber $D^\textrm{b}(X)$. It is proved (cf. [10, 11, 21]) that the morphism (1.1) maps the simple loops around the points $\infty , 1$ to the autoequivalences  and , respectively, where we put , and  denotes the spherical twist around the structure sheaf. Note that by taking the cohomology group of *X*, and restricting it to the subring $\Lambda _H(X) \subset H^*(X, \mathbb {Q})$ generated by the hyperplane class, the homomorphism (1.1) induces a usual local system *L* with the fiber $\Lambda _H(X)$:1.2There is a canonical way to extend *L* as a perverse sheaf on $\mathbb {P}^1$, called the *intersection complex* and denoted by $\textrm{IC}(L)$.

The aim of this paper is to give a categorification of the intersection complex $\textrm{IC}(L)$:

#### Theorem 1.1

(Theorems 5.7 and 6.7) There exists a perverse schober ${\mathfrak P}$ on $\mathbb {P}^1$ which extends the local system (1.1) of categories. More precisely, the perverse schober ${\mathfrak P}$ is a categorification of the intersection complex $\textrm{IC}(L)$ associated to the local system (1.2).

A computation shows that the intersection complex $\textrm{IC}(L)$ has a (anti-) symmetric pairing. In particular, it is Verdier self-dual. In Proposition 5.9, we will also prove that our perverse schober ${\mathfrak P}$ has a categorification of this property introduced in [25], see Definition 3.12 and Remark 3.13.

In the case of elliptic curves, we also construct a perverse schober ${\mathfrak P}^A$ on $\mathbb {P}^1$ from the *A*-side and prove:

#### Theorem 1.2

(Theorem 7.3) Let *X* be an elliptic curve, $X^\vee$ its mirror. Then the perverse schober ${\mathfrak P}^A$ on $\mathbb {P}^1$ has a generic fiber $D^\pi \textrm{Fuk}(X^\vee )$, and it is identified with the perverse schober ${\mathfrak P}$ in Theorem 1.1 under the mirror equivalence $D^\textrm{b}(X) \simeq D^\pi \textrm{Fuk}(X^\vee )$.

#### Remark 1.3

Our definition of perverse schobers is slightly more general than the original definition given in [24], see Definition 3.4 and Example 3.6. This generalization is essential for our construction.

### Idea of proof

To construct the perverse schober as in Theorem 1.1, we need to find spherical functors which induce the autoequivalences , and . For the first two autoequivalences, we can find natural spherical functors using the derived categories of varieties.

For the last equivalence , we use the following Orlov equivalence:

$$\begin{aligned} D^\textrm{b}(X) \simeq \textrm{HMF}^{\,\mathrm gr}(W), \end{aligned}$$

to find the natural spherical functor, where *W* is the homogeneous polynomial defining $X \subset \mathbb {P}^{\,n+1}$. We find that the autoequivalence  has a natural presentation as the twist of a spherical functor between categories of graded matrix factorizations.

Note that, for a given autoequivalence, there are many ways to express it as a twist of a spherical functor. We choose the natural expressions so that we recover the intersection complex after decategorification.

### Related works

Various examples of perverse schobers, consisting of derived categories of varieties, have been constructed using birational geometry [9, 12, 13, 34, 35]. Our perverse schober has a different origin from these examples, in the sense that it does not involve birational geometric structure. Moreover, it is the first example involving a Landau–Ginzburg model, which defines the category of graded matrix factorizations.

Donovan–Kuwagaki [14] verified a mirror symmetry type statement for some examples of perverse schobers coming from non-compact geometry, including the case of Atiyah flops. Theorem 1.2 is an analogue of their result, for the case of elliptic curves.

### Open questions

1. It would be interesting to construct a perverse schober using Fukaya categories, which is mirror to our schober in higher dimension. For example, in the case of a quartic K3 surface, we need to understand mirror symmetry for the categories $D^\textrm{b}(C)$ and , where *C* is a smooth projective curve of genus 3, and *W* is a homogeneous polynomial of degree 4.
2. In the case of a quartic K3 surface, we can think of the Riemann sphere $\mathbb {P}^1$ as a compactification of a certain quotient of the space of normalized Bridgeland stability conditions. It would be interesting to generalize our construction to arbitrary K3 surfaces.

### Plan of the paper

The paper is organized as follows: In Sect. 2, we review the theory of dg categories used in this paper. In particular, we recall the notion of spherical dg functors. In Sect. 3, we define the notion of perverse schobers on a Riemann surface following Kapranov–Schechtman [24]. In Sect. 4, we review the theory of derived factorization categories. In particular, we recall various constructions of dg enhancements of these categories.

In Sect. 5, we construct our perverse schober. In Sect. 6, we prove that our perverse schober categorifies the intersection complex. In Sect. 7, we discuss the mirror symmetry of perverse schobers for elliptic curves. Finally in Sect. 8, we review the proof of Orlov equivalences via a variation of GIT quotients, following [5]. Using this, we construct an example of a spherical pair, which is another categorification of a perverse sheaf on a disk.

**Notation and convention** Throughout the paper, we work over the complex number field $\mathbb {C}$.

- For a variety *X*, $D^\textrm{b}(X)$ denotes the bounded derived category of coherent sheaves on *X*.
- For an integer $d \in \mathbb {Z}$,  denotes the character defined by .

## Quick review on dg categories

In this section, we briefly review the theory of dg categories used in this paper. We refer to [1, 26, 27, 36, 37] for more details.

### Basic definitions

We denote by ${{\,\textrm{dgcat}\,}}_{\,\mathbb {C}}$ the category of small dg categories over $\mathbb {C}$. For a dg category , we denote by  the *homotopy category* of .

#### Definition 2.1

Let  be dg categories, and let  be a dg functor. We say that the functor *F* is a *quasi-equivalence* if it satisfies the following two conditions:

1. For any objects , the morphism  is a quasi-isomorphism.
2. The induced functor  on the homotopy categories is essentially surjective.

We denote by ${{\,\textrm{hodgcat}\,}}_{\,\mathbb {C}}$ the localization of ${{\,\textrm{dgcat}\,}}_{\,\mathbb {C}}$ by quasi-equivalences. A morphism in ${{\,\textrm{hodgcat}\,}}_{\,\mathbb {C}}$ is called a *quasi-functor*.

#### Dg modules and derived categories

We denote by ${{\,\textrm{Mod}\,}}_{\,\mathbb {C}}$ the dg category of complexes of $\mathbb {C}$-vector spaces. Let  be a dg category. A *right* *-module* is a dg functor . We denote by  the dg category of right -modules. We have the *dg Yoneda embedding*2.1

##### Definition 2.2

1. An object  is *acyclic* if for every , the complex $C(a) \in {{\,\textrm{Mod}\,}}_{\,\mathbb {C}}$ is acyclic.
2. An object  is *projective* if for every acyclic module , we have .

We denote by  the dg subcategory consisting of projective modules. The *derived category*  is the localization of the homotopy category  by acyclic -modules. The derived category  has the structure of a triangulated category, and we have a canonical equivalence .

We denote by  the full triangulated subcategory consisting of compact objects. A right -module is called *perfect* if its class in the derived category  is compact. We denote by  the dg subcategory of perfect projective modules. Note that we have an equivalence .

The dg Yoneda embedding (2.1) induces the embeddingNote that the homotopy category  is not triangulated in general. We define the triangulated category  to be the smallest triangulated subcategory containing . Then we have the following inclusions:

##### Definition 2.3

A dg category  is *pre-triangulated* (resp. *triangulated*) if the inclusion  (resp. ) is an equivalence.

##### Remark 2.4

A pre-triangulated dg category  is triangulated if and only if its homotopy category  is idempotent complete (cf. [27, Theorem 3.8]).

#### Bimodules

Let  be dg categories. An -*-bimodule* is an -module. We denote by  the dg category of --bimodules, and by  its derived category.

##### Definition 2.5

Let *M* be an --bimodule. We say that *M* is *-perfect* (resp. *-perfect*) if  (resp. ) is perfect for all  (resp. ).

We denote by  (resp. ) the full triangulated subcategory of  consisting of -perfect (resp. -perfect) bimodules.

Given a bimodule , we have the *tensor product* functorand its derived functorSimilarly, we have the functorsWe have the following characterizations of -perfect and -perfect bimodules (see the first and second paragraphs in [1, p. 2590]):

- *M* is -perfect if and only if the derived tensor product  restricts to the functor .
- *M* is -perfect if and only if the derived tensor product  restricts to the functor .

### Dg enhancements

We first define the notion of *dg enhancements*:

#### Definition 2.6

Let  be a triangulated category.

1. A *dg enhancement* of  is a pair  consisting of a pre-triangulated dg category  and an exact equivalence .
2. A *Morita enhancement* of  is a pair  consisting of a dg category  and an exact equivalence .

#### Remark 2.7

Suppose that a dg category  is triangulated. If we have a dg enhancement  of a triangulated category , it also gives a Morita enhancement via

#### Example 2.8

Let  be a dg category. The dg category  is triangulated and gives a Morita enhancement of .

For our purpose, we also need the notion of enhancements of exact functors of triangulated categories.

#### Theorem 2.9

([36]) Let  be dg categories. There exists a dg category  with the following properties:

1. There exists a bijection  where the left-hand side denotes the set of isomorphism classes of objects in .
2. There exists an equivalence  such that for an object , the corresponding exact functor  is .

#### Proof

The existence of the dg category  is proved in [36, Theorem 6.1]. The first assertion is [36, Corollary 4.8]; the second assertion is proved in [36, Theorem 7.2]. $\square$

#### Definition 2.10

Let  be an exact functor of triangulated categories. Suppose that the triangulated categories  have dg enhancements , respectively. Then a *dg enhancement* of the functor $\Phi$ is an object  together with an isomorphism .

### Spherical functors

Let  be dg categories, let  be an -perfect and -perfect bimodule. Recall from Theorem 2.9 (2) that *S* defines an isomorphism class of quasi-functors  whose underlying exact functor  is isomorphic to the derived tensor product . We denote as2.2By [1, Corollary 2.2], there exist -perfect and -perfect objects  such that the functorsare the left, right adjoints of the functor (2.2), respectively.

Let us denote by *SR* the object . We define objects ,  in a similar way. Then the corresponding derived tensor products are isomorphic to the functors *sr*, *sl*, *rs*, *ls*, respectively. By [1, Definitions 2.3, 2.4], there exist morphismswhich induce adjoint (co)units on the underlying exact functors. Note that we regard  as *diagonal* --bimodule, --bimodule, respectively. Namely, we define  asand similarly for . By taking (shifts of) cones, we obtain the exact trianglesWe call *T* (resp. ) as *twist* (resp. *dual twist, cotwist, dual cotwist*) of *S*. We denote by  their underlying exact functors.

The following is the main result of [1]:

#### Definition-Theorem 2.11

([1, Theorem 5.1]) Suppose that any two of the following conditions hold:

- *t* is an autoequivalence of .
- *f* is an autoequivalence of .
- The composition $lt[-1] \rightarrow lsr \rightarrow r$ is an isomorphism of functors.
- The composition $r \rightarrow rsl \rightarrow fl[1]$ is an isomorphism of functors.

Then all four hold. If this is the case, we call the object  *spherical*.

The following result will be useful for our purpose:

#### Theorem 2.12

([6, Theorem B]) Let  be small dg categories. Suppose that we have spherical functors  and  whose twists are  and , respectively.

Then there exists a dg category  with a semi-orthogonal decomposition , and a spherical functor  whose twist is .

## Local systems of categories and perverse schobers

In this section, we recall the notion of perverse schobers on Riemann surfaces, which categorifies perverse sheaves. We refer to [12, 13, 24] for the details.

### Local systems of categories

#### Definition 3.1

Let *M* be a manifold and let $Z \subset M$ be a finite subset. We define a *Z-coordinatized local system of categories* to be an action of the fundamental groupoid $\pi _1(M, Z)$, i.e., it consists of the following datum:

- a category  for each $z \in Z$,
- an equivalence  for each path $g \in \pi _1(M, Z)$,
- a natural isomorphism $\theta _{g, h} :\rho _g \rho _h \rightarrow \rho _{gh}$ for each pair (*g*, *h*) of composable paths,

such that the diagrams3.1commute for all composable paths $g, h, k \in \pi _1(M, Z)$.

### Quiver description of perverse sheaves on a disk

To motivate the definition of perverse schobers, we first recall the quiver description of the category of usual perverse sheaves on a disk.

Let $\Delta$ be the unit disk, $B=\{b_1, \dots , b_n \} \subset \Delta$ a finite subset. We denote by $\textrm{Perv}(\Delta , B)$ the category of perverse sheaves on $\Delta$ singular at *B* (i.e., whose restrictions to  are local systems).

#### Definition 3.2

We define  to be the category of data $(D, D_i, u_i, v_i)_{i=1}^n$ consisting of finite-dimensional vector spaces $D, D_i$ and linear maps $u_i :D \rightarrow D_i$, $v_i :D_i \rightarrow D$ such that the endomorphisms  are isomorphisms for all $i = 1, \dots , n$.

Fix a point $p \in \partial \Delta$. A *skeleton* is a union of simple arcs joining *p* and $b_i$ for all $b_i \in B$, coinciding near *p*. Let  be the set of isotopy classes of skeletons. We will see that there are equivalences between the categories $\textrm{Perv}(\Delta , B)$ and  parametrized by the set , compatible with the natural action of the Artin braid groupFor a generator $s_i \in {{\,\textrm{Br}\,}}_n$, we define an autoequivalence  as follows: For an object , we definewhere3.2For a general element $\sigma \in {{\,\textrm{Br}\,}}_n$, we then define an equivalence  as the composition of $f_{s_i}$’s and their inverses.

#### Proposition 3.3

([17]) For each class , there is an equivalenceof categories, such that, for every $\sigma \in {{\,\textrm{Br}\,}}_n$, the following diagram commutes:

### Perverse schobers on Riemann surfaces

First we define the notion of perverse schobers on a disk. We keep the notations in the previous subsection.

#### Definition 3.4

([13, 24])

1. A *coodinatized schober* on $(\Delta , B)$ is a datum  consisting of triangulated dg categories , spherical functors , and numbers $\epsilon _i \in \{\pm 1\}$. For each *i*, we denote by $R_i, L_i$ the right and left adjoints of $S_i$, and by  (resp. ) the corresponding twist (resp. dual twist) equivalence.
2. For a given coordinatized schober ${\mathfrak S}$ and an element $\sigma \in {{\,\textrm{Br}\,}}_n$, we define a new coordinatized schober $f_\sigma ({\mathfrak S})$ as in (3.2), replacing $(D, D_i, u_i, v_i)$ with
  (resp. ), and endomorphism $T_i={{\,\textrm{id}\,}}-v_iu_i$ with the functor $T_i$ (resp. $T'_i$) when $\epsilon _i=1$ (resp. $\epsilon _i=-1$).
3. A *perverse schober* on $(\Delta , B)$ is a collection  of coordinatized schobers $\mathfrak {S}_K$ such that for each $\sigma \in {{\,\textrm{Br}\,}}_n$, we have a compatible identification
  $$\begin{aligned} f_{\sigma } {\mathfrak S}_K \xrightarrow { \ \sim \ } {\mathfrak S}_{\sigma (K)}. \end{aligned}$$

#### Remark 3.5

By [1, Proposition 5.3], we have an isomorphism $T'_i \cong T_i^{-1}$ for each *i*.

A choice of signs $\epsilon _i$ in the above definition is important when we consider decategorifications:

#### Example 3.6

Let *X* be a smooth projective Calabi–Yau variety of dimension *n*. Let $E \in D^\textrm{b}(X)$ be a spherical object, e.g., . Then we obtain a spherical functorDenote by *L*, *R* its left and right adjoints.

Taking the cohomology groups, we get the following diagrams:3.3where  denote the cohomological Fourier–Mukai transforms. Since the canonical bundles of ${{\,\textrm{pt}\,}}$ and *X* are trivial, we have . Hence if *n* is odd, the diagrams (3.3) define two different perverse sheaves on a disk (see Definition 3.2).

#### Definition 3.7

Let  be a coordinatized perverse schober on $(\Delta , B)$. We define an *induced local system* of categories on $\partial \Delta$ to be the one whose monodromy autoequivalence is

We now define the notion of perverse schobers on Riemann surfaces. Let $\Sigma$ be a Riemann surface and $B \subset \Sigma$ a finite set of points. Take a disk $\Delta \subset \Sigma$ containing the set *B*. Denote by *U* the closure of .

We fix a point $p \in \partial \Delta$ and a finite set . We set .

#### Definition 3.8

([13, 24]) A *perverse schober* on $(\Sigma , B)$ is a datum consisting of

- a perverse schober ${\mathfrak S}_{\Delta }$ on $(\Delta , B)$,
- a *Z*-coordinatized local system ${\mathfrak L}$ of categories on *U*,

such that the induced $\{p\}$-coordinatized local systems ${\mathfrak S}_\Delta |_{\partial \Delta }$ and ${\mathfrak L}|_{\partial \Delta }$ are isomorphic.

#### Example 3.9

Consider the case $\Sigma =\mathbb {P}^1$. In this case, giving a perverse schober on  is equivalent to giving a perverse schober on $(\Delta , B)$ such that the induced local system on $\partial \Delta$ has the trivial monodromy.

The following result is very useful for our purpose:

#### Proposition 3.10

([13, Proposition 4.10]) Fix a point . Let ${\mathfrak S}_{\Sigma \setminus \{p\}}$ be a schober on ,  be the corresponding monodromy autoequivalence around the point *p*.

Given a presentation of the equivalence *T* as the twist or the dual twist of a spherical functor, we obtain a schober on $(\Sigma , B \,{\cup }\, \{p\} )$.

#### Remark 3.11

Note that there are many ways presenting an equivalence *T* as a spherical (dual) twist, and a schober on $\Sigma$ in the above proposition depends on such a choice. We will find the most natural presentations in our setting.

Finally we recall the notion of Calabi–Yau perverse schobers:

#### Definition 3.12

([25, Definitions 3.1.8 and 3.1.14]) Let $n \in \mathbb {Z}_{>0}$ be a positive integer.

1. A perverse schober on $(\Delta , 0)$, represented by a spherical functor , is *n-Calabi–Yau* (CY) if the following conditions hold:
  - The category  is CY of dimension *n*,
  - The shifted cotwist $f[n+1]$ is the Serre functor of .
2. A perverse schober ${\mathfrak S}$ on $(\Sigma , B)$ is *n-Calabi–Yau* if the following conditions hold:
  - The restriction ${\mathfrak S}|_{\Sigma \setminus B}$ is a local system of *n*-CY categories, and the monodromy autoequivalences preserve the CY structure,
  - For each point $b \in B$, the restriction of ${\mathfrak S}$ to a disk around *b* is *n*-CY.

#### Remark 3.13

The notion of *n*-CY perverse schobers categorifies perverse sheaves with (anti-)symmetric structures with respect to the Verdier duality, see [25, Proposition 1.3.22]. In particular, these perverse sheaves are Verdier self-dual.

### Spherical pairs

Consider the special case where $\Sigma =\Delta$ and $B=\{0\}$. In this case, we have another categorification of perverse sheaves on $(\Delta , 0)$, called *spherical pair*:

#### Definition 3.14

A *spherical pair* is a pair of semi-orthogonal decompositionsof a triangulated category  such that the compositions of the inclusions and the projectionsare equivalences.

#### Remark 3.15

As proved in [24, Proposition 3.7], given a spherical pair, we obtain a spherical functor . Hence a spherical pair induces a perverse schober on $(\Delta , 0)$.

As we will see in Sect. 8, the theory of variations of GIT quotients, developed by [5, 18], provides natural classes of spherical pairs.

## Derived factorization category

### Derived factorization category

In this subsection, we recall the notion of derived factorization categories following [4].

Let *G* be an affine algebraic group acting on a variety *X*, and $\chi :G \rightarrow \mathbb {C}^*$ be a character of *G*. Let $W:X\rightarrow \mathbb {C}$ be a $\chi$-semi-invariant regular function, i.e.  for any $g\in G$ and any $x\in X$. The data $(X,\chi ,W)^G$ is called a *gauged Landau–Ginzburg model*. Denote the character invertible sheaf of $\chi$ on *X* by . First, we define the dg category ${{\,\textrm{fact}\,}}_G(X,\chi ,W)$ of factorizations of $(X,\chi ,W)^G$.

#### Definition 4.1

A *factorization* of $(X,\chi ,W)^G$ is a sequence

4.1 $$\begin{aligned} E=\bigl (E_1\xrightarrow { \ \varphi _1^E \ } E_0\xrightarrow { \ \varphi _0^E \ } E_1(\chi )\bigr ) \end{aligned}$$

of morphisms of *G*-equivariant coherent sheaves on *X* such thatA factorization $E=\bigl (E_1\xrightarrow {\varphi _1^E} E_0\xrightarrow {\varphi _0^E} E_1(\chi )\bigr )$ of $(X,\chi ,W)^G$ is called a *locally free factorization* if $E_1$ and $E_0$ are *G*-equivariant locally free sheaves on *X*.

#### Definition 4.2

Let

$$\begin{aligned} E=\bigl (E_1\xrightarrow { \ \varphi _1^E \ } E_0\xrightarrow { \ \varphi _0^E \ } E_1(\chi )\bigr ),\quad F=\bigl (F_1\xrightarrow { \ \varphi _1^F \ } F_0\xrightarrow { \ \varphi _0^F \ } F_1(\chi )\bigr ) \end{aligned}$$

be factorizations of $(X,\chi ,W)^G$.

We define the $\mathbb {Z}$-graded $\mathbb {C}$-vector space ${{\,\textrm{Hom}\,}}_{\,{{\,\textrm{fact}\,}}_G(X,\chi ,W)}(E,F)$ of morphisms from *E* to *F* as follows. For an integer *l*, we defineThen we defineMoreover, we define the linear map

$$\begin{aligned} d_{E,F}:{{\,\textrm{Hom}\,}}_{\,{{\,\textrm{fact}\,}}_G(X,\chi ,W)}(E,F) \rightarrow {{\,\textrm{Hom}\,}}_{\,{{\,\textrm{fact}\,}}_G(X,\chi ,W)}(E,F) \end{aligned}$$

as follows. Take $f=(f_1,f_0) \in {{\,\textrm{Hom}\,}}^{n}_{\,{{\,\textrm{fact}\,}}_G(X,\chi ,W)}(E,F)$. When $n=2\,l$ for some integer *l*, we putWhen $n=2l+1$ for some integer *l*, we putThen $d_{E,F}^2=0$ holds and the degree of $d_{E,F}$ is one. We have a dg $\mathbb {C}$-module $({{\,\textrm{Hom}\,}}_{\,{{\,\textrm{fact}\,}}_G(X,\chi ,W)}(E,F), d_{E,F})$.

Thus, we have the dg category of factorizations of $(X,\chi ,W)^G$.

#### Definition 4.3

Denote the dg category of factorizations of $(X,\chi ,W)^G$ by ${{\,\textrm{fact}\,}}_G(X,\chi ,W)$. Let ${{\,\textrm{vect}\,}}_G(X,\chi ,W)$ be the dg subcategory of locally free factorizations of $(X,\chi ,W)^G$.

We obtain triangulated categories from the dg categories in Definition 4.3.

#### Proposition 4.4

([4, Proposition 3.7]) The dg categories ${{\,\textrm{fact}\,}}_G(X,\chi ,W)$ and $\textrm{vect}_G(X,\chi ,W)$ are triangulated dg categories.

Next, we define the derived factorization categories $\textrm{D}^{\textrm{abs}}{{\,\textrm{fact}\,}}_G(X,\chi ,W)$ and $\textrm{D}^{\textrm{abs}}[{{\,\textrm{fact}\,}}_G(X,\chi ,W)]$ of $(X,\chi ,W)^G$.

#### Remark 4.5

The category $Z^0({{\,\textrm{fact}\,}}_G(X,\chi ,W))$ has the structure of an abelian category, where the objects of $Z^0({{\,\textrm{fact}\,}}_G(X,\chi ,W))$ are same as ${{\,\textrm{fact}\,}}_G(X,\chi ,W)$. For objects $E,F \in Z^0({{\,\textrm{fact}\,}}_G(X,\chi ,W))$, we define .

To introduce the notion of acyclic objects, we need the totalization of an object in $Z^0({{\,\textrm{fact}\,}}_G(X,\chi ,W))$.

#### Definition 4.6

Let  be a complex of objects in the abelian category $Z^0({{\,\textrm{fact}\,}}_G(X,\chi ,W))$. For $i \in \mathbb {Z}$, we put

$$\begin{aligned} E^i=\bigl (E^i_1\xrightarrow { \ \varphi _1^{E^i}} E^i_0\xrightarrow {\ \varphi _0^{E^i}} E^i_1(\chi )\bigr ). \end{aligned}$$

We define *G*-equivariant quasi-coherent sheaves $T_1, T_0$ on *X* asWe define morphisms $t_1, t_0$ of *G*-equivariant quasi-coherent sheaves asThe *totalization*  of  is an object defined as

#### Definition 4.7

A factorization $E \in {{\,\textrm{fact}\,}}_G(X,\chi ,W)$ is called *acyclic* if there is an acyclic complex  of objects in $Z^0({{\,\textrm{Fact}\,}}_G(X,\chi ,W))$ such that *E* is isomorphic to  in $Z^0({{\,\textrm{fact}\,}}_G(X,\chi ,W))$. Let ${{\,\textrm{acyc}\,}}_G(X,\chi ,W)$ be the full dg subcategory of acyclic factorizations in ${{\,\textrm{fact}\,}}_G(X,\chi ,W)$.

#### Definition 4.8

We define the *absolute derived category* $D^{\textrm{abs}}[{{\,\textrm{fact}\,}}_G(X,\chi ,W)]$ of $[{{\,\textrm{fact}\,}}_G(X,\chi ,W)]$ to be the Verdier quotient

#### Definition 4.9

We define the *absolute derived category* $D^{\textrm{abs}}{{\,\textrm{fact}\,}}_G(X,\chi ,W)$ of ${{\,\textrm{fact}\,}}_G(X,\chi ,W)$ to be the dg quotient

The categories $\textrm{D}^{\textrm{abs}}{{\,\textrm{fact}\,}}_G(X,\chi ,W)$ and $\textrm{D}^{\textrm{abs}}[{{\,\textrm{fact}\,}}_G(X,\chi ,W)]$ are also called the *derived factorization categories* of $(X,\chi ,W)^G$.

#### Proposition 4.10

([4, Corollary 5.9, Proposition 5.11]) The dg category $D^{\textrm{abs}}{{\,\textrm{fact}\,}}_G(X,\chi ,W)$ is a dg enhancement of $D^{\textrm{abs}}[{{\,\textrm{fact}\,}}_G(X,\chi ,W)]$. If *X* is smooth affine and *G* is reductive, the canonical dg functor

$$\begin{aligned} {{\,\textrm{vect}\,}}_G(X,\chi ,W) \rightarrow D^{\textrm{abs}}{{\,\textrm{fact}\,}}_G(X,\chi ,W) \end{aligned}$$

is a quasi-equivalence.

### Derived functors

In this subsection, we recall the construction of derived functors which we will use later. Let *X* and *Y* be smooth affine varieties, and *G* a reductive affine algebraic group acting on *X* and *Y*. Let $f:X \rightarrow Y$ be a *G*-equivariant morphism. Take a character $\chi :G \rightarrow \mathbb {C}^*$ of *G*. Let $W:Y \rightarrow \mathbb {C}$ be a $\chi$-semi-invariant regular function on *Y*. Then $(Y,\chi ,W)^G$ and $(X,\chi ,f^*W)^G$ are gauged Landau–Ginzburg models.

#### Definition 4.11

The dg functor $f^*:{{\,\textrm{vect}\,}}_G(Y,\chi ,W) \rightarrow {{\,\textrm{vect}\,}}_G(X,\chi ,f^*W)$ is defined as follows. For an object $E \in {{\,\textrm{vect}\,}}_G(Y,\chi ,W)$, we define an objectFor a morphism $p=(p_1,p_0) \in {{\,\textrm{Hom}\,}}_{{{\,\textrm{vect}\,}}_G(Y,\chi ,W)}(E,F)$, we define a morphismBy Proposition 4.10, we have the exact functor

$$\begin{aligned} D^{\textrm{abs}}[{{\,\textrm{fact}\,}}_G(Y,\chi ,W)]\rightarrow D^{\textrm{abs}}[{{\,\textrm{fact}\,}}_G(X,\chi ,f^*W)] \end{aligned}$$

and denote it by $f^*$.

#### Definition 4.12

Assume that *f* is a closed immersion. We define the dg functor $f_*:{{\,\textrm{fact}\,}}_G(X,\chi ,f^*W) \rightarrow {{\,\textrm{fact}\,}}_G(Y,\chi ,W)$ as follows. For an object $E \in {{\,\textrm{fact}\,}}_G(X,\chi ,f^*W)$, we define an objectFor a morphism $p=(p_1,p_0) \in {{\,\textrm{Hom}\,}}_{{{\,\textrm{fact}\,}}_G(X,\chi ,f^*W)}(E,F)$, we define a morphismSince *f* is a closed immersion, $f:{{\,\textrm{fact}\,}}_G(X,\chi ,f^*W) \rightarrow {{\,\textrm{fact}\,}}_G(Y,\chi ,W)$ sends acyclic factorizations to acyclic factorizations. Therefore, we obtain the dg functor

$f_*:\textrm{D}^{\textrm{abs}}{{\,\textrm{fact}\,}}_G(X,\chi ,f^*W) \rightarrow \textrm{D}^{\textrm{abs}}{{\,\textrm{fact}\,}}_G(Y,\chi ,W)$ and the exact functor $f_*:\textrm{D}^{\textrm{abs}}[{{\,\textrm{fact}\,}}_G(X,\chi ,f^*W)]\rightarrow \textrm{D}^{\textrm{abs}}[{{\,\textrm{fact}\,}}_G(Y,\chi ,W)]$.

#### Definition 4.13

Let $(X,\chi ,W)^G$ be a gauged Landau–Ginzburg model. Let *L* be a *G*-equivariant line bundle on *X*. We define the dg functor  as follows. For an object $E \in {{\,\textrm{fact}\,}}_G(X,\chi ,W)$, we define an objectFor a morphism $f=(f_1,f_0) \in {{\,\textrm{Hom}\,}}_{{{\,\textrm{fact}\,}}_G(X,\chi ,W)}(E,F)$, we define a morphismRestricting this dg functor to the full dg subcategory ${{\,\textrm{vect}\,}}_G(X,\chi ,W)$, we obtain the dg functor . Note that it induces the autoequivalence

More generally, for , we have the derived tensor functorby [20, Proposition 4.23]. By [20, Definition 3.14], there is the exact functor $\Upsilon :D^\textrm{b}({{\,\textrm{Coh}\,}}_G(X)) \rightarrow \textrm{D}^{\textrm{abs}}[{{\,\textrm{fact}\,}}_G(X,\chi ,0)]$ sending a *G*-equivariant coherent sheaf *A* to the factorization $(0 \,{\rightarrow }\, A \,{\rightarrow }\, 0)$. For simplicity, we also denote the composition  by  as in [20, Definition 4.24].

### Koszul factorizations

Let $(X, \chi , W)^G$ be a gauged Landau–Ginzburg model. Assume that *X* is smooth. Let  be a *G*-equivariant locally free sheaf on *X* of finite rank. Let  and  be morphisms of *G*-equivariant coherent sheaves such that  and . Let $Z_s \subset X$ be the zero scheme of the section . The section *s* is called regular if the codimension of $Z_s$ in *X* is equal to the rank of .

#### Definition 4.14

We define the *Koszul factorization* $K(s,t) \in {{\,\textrm{vect}\,}}_G(X,\chi ,W)$ of *s* and *t* aswhereand

We will use the following lemma later:

#### Lemma 4.15

([4, Proposition 3.20, Lemma 3.21]) The following statements hold:

1. There is the natural isomorphism
2. If *s* is regular, there are the natural isomorphisms  in $\textrm{D}^{\textrm{abs}}[{{\,\textrm{fact}\,}}_G(X,\chi ,W)]$

### Knörrer periodicity

Let *X* be a smooth quasi-projective variety. Consider the trivial action of $\mathbb {C}^*$ on *X*. Let  be a $\mathbb {C}^*$-equivariant locally free sheaf on *X* of finite rank. Take a $\mathbb {C}^*$-invariant regular section . Then we have the $\chi _1$-semi-invariant regular function  induced by *s*. Let  be the $\mathbb {C}^*$-equivariant vector bundle on *X*. Take the restriction  of the $\mathbb {C}^*$-equivariant vector bundle *q* to $Z_s$. Then there is the following commutative diagram:Shipman [33] proved the following theorem. See also [22, 20, Theorem 4.1].

#### Theorem 4.16

([33, Theorem 3.4]) We have the equivalence4.2

The equivalence in Theorem 4.16 is called the *Knörrer periodicity*. By [20, Proposition 2.14], there is the canonical equivalence

4.3 $$\begin{aligned} D^\textrm{b}(Z_s) \xrightarrow { \ \sim \ } \textrm{D}^{\textrm{abs}}[{{\,\textrm{fact}\,}}_{\mathbb {C}^*}(Z_s,\chi _1,0)]. \end{aligned}$$

We also denote by  the composition of the equivalences (4.2) and (4.3).

### Graded matrix factorizations

Let  be the polynomial ring. We regard $S_n$ as a graded $\mathbb {C}$-algebra, where the degree of $x_i$ is one for each $i = 1, \dots , n$. Let  be the category of finitely generated graded $S_n$-modules whose morphisms are degree preserving homomorphisms. Let  be the full subcategory of finitely generated graded free $S_n$-modules.

We recall the definition of graded matrix factorizations. Fix a positive integer $d \in \mathbb {Z}_{>0}$. Let $W \in S_n$ be a homogeneous polynomial of degree *d*.

#### Definition 4.17

A *graded matrix factorization* of *W* is a sequence

$$\begin{aligned} M=\bigl (M_1\xrightarrow {\varphi _1^M} M_0\xrightarrow {\varphi _0^M} M_1(d)\bigr ), \end{aligned}$$

where $M_1$ and $M_0$ are finitely generated free graded $S_n$-modules and $\varphi _1^M$ and $\varphi _0^M$ are degree preserving homomorphisms such that

As similar to Definitions 4.2 and 4.3, we can define the *dg category* ${{\,\mathrm{MF^{\,gr}}\,}}(W)$ *of graded matrix factorizations of* *W*. Let $\textrm{HMF}^{\,\mathrm gr}(W)$ be the homotopy category $[{{\,\mathrm{MF^{\,gr}}\,}}(W)]$. For an integer $l \in \mathbb {Z}$ and a graded $S_n$-module $P=\bigoplus _{i \in \mathbb {Z}} P_i$, we define the graded $S_n$-module $P(l)=\bigoplus _{i \in \mathbb {Z}}P(l)_i$ by . Then we obtain the degree shift functors  and $(l):{{\,\mathrm{MF^{\,gr}}\,}}(W) \xrightarrow {\sim } {{\,\mathrm{MF^{\,gr}}\,}}(W)$. We put .

### Matrix factorizations and Landau–Ginzburg models

Fix a positive integer *d* and a homogeneous polynomial $W \in S_n$ of degree *d*. Consider the action of $\mathbb {C}^*$ on the affine space $\mathbb {C}^{n}$ defined byfor $\lambda \in \mathbb {C}^*$ and $(x_1, \dots , x_n) \in \mathbb {C}^n$. Let  be the character of $\mathbb {C}^*$ defined by . Then  is a $\chi _d$-semi-invariant regular function. The data  is a gauged Landau–Ginzburg model.

Denote the category of $\mathbb {C}^*$-equivariant locally free coherent sheaves on $\mathbb {C}^n$ by ${{\,\textrm{vect}\,}}_{\,\mathbb {C}^*}(\mathbb {C}^n)$. Taking global sections, we have the equivalence . Note that, for $E \in {{\,\textrm{vect}\,}}_{\,\mathbb {C}^*}(\mathbb {C}^n)$, the $S_n$-module $\Gamma (E)$ has the structure of a graded $S_n$-module induced by the weight decomposition with respect to the induced action of $\mathbb {C}^*$ on $\Gamma (E)$. This induces the equivalence  of dg categories. Hence by Proposition 4.10, we have the quasi-equivalence . Taking homotopy categories, we obtain the equivalence

#### Remark 4.18

By [29, Theorem 40] and [2, Lemma 4.8], the triangulated category $\textrm{HMF}^{\,\mathrm gr}(W)$ is idempotent complete.

### Serre functor

Fix a positive integer *d* and a homogeneous polynomial $W \in S_n$ of degree *d*. The Serre functor of $\textrm{HMF}^{\,\mathrm gr}(W)$ is described as follows.

#### Theorem 4.19

([23, Theorem 3.8]) The Serre functor *S* of $\textrm{HMF}^{\,\mathrm gr}(W)$ is given by .

Using Theorem 4.19, we describe the Serre functor of . Note that the following diagram commutes:Taking homotopy categories, we obtain the commutative diagram:4.4By Theorem 4.19 and (4.4), we have the following.

#### Theorem 4.20

([23, Theorem 3.8]) The functoris the Serre functor.

## Construction of perverse schobers

Let $n \geqslant 1$ be a positive integer, $W \in \mathbb {C}[x_1, \dots , x_{n+2}]$ be a homogeneous polynomial of degree $n+2$. We take the polynomial *W* general so that the hypersurface  is smooth, which is a projective Calabi–Yau variety of dimension *n*. We denote by .

In this section, we construct a perverse schober using the following Orlov’s theorem:

### Theorem 5.1

([29, 3, Proposition 5.8]) There exists an equivalence

$$\begin{aligned} \psi :D^\textrm{b}(X) \xrightarrow { \ \sim \ } \textrm{HMF}^{\,\mathrm gr}(W) \end{aligned}$$

between triangulated categories. Moreover, the following diagram commutes:5.1

### Remark 5.2

More precisely, we have equivalences

$$\begin{aligned} \psi _w :D^\textrm{b}(X) \rightarrow \textrm{HMF}^{\,\mathrm gr}(W) \end{aligned}$$

indexed by integers $w \in \mathbb {Z}$, and we have the commutative diagram (5.1) for a particular choice of $w \in \mathbb {Z}$. See Sect. 8 for more detail.

Take a disk  containing the point 1. We fix two distinct points . First we construct a $\{x^\pm \}$-coordinatized local system of categories on .

**Fig. 1:**
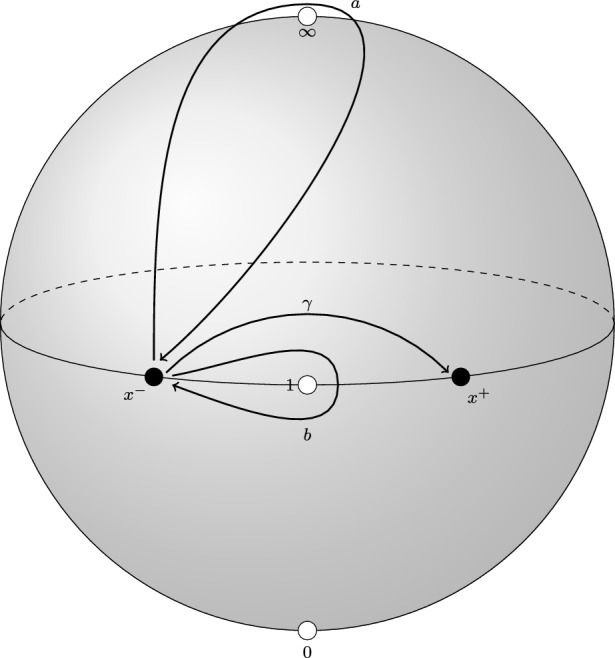
Generators of the groupoid

Let  be simple loops around $\infty , 1$, respectively. Let $\gamma$ be a path from $x^-$ to $x^+$ which is contained in $\Delta$ and does not go around the point $1 \in \Delta$. See Fig. 1 below. Then the groupoid  is freely generated by the three paths $a, b, \gamma$.

We first prepare the following two lemmas:

### Lemma 5.3

There is a $\{x^-\}$-coordinatized local system ${\mathfrak K}$ on  with assignments

### Proof

Consider a homomorphism5.2which sends the generators *a*, *b* to the autoequivalences , ${{\,\textrm{ST}\,}}_{\mathscr {O}_X}$, respectively.

By [7, Theorem 2.1 (1)] (see also Section 2.3 in the same paper), there exists an obstruction class $o \in H^3(G, \mathbb {C}^*)$ for lifting the homomorphism (5.2) to an action of *G* on the category $D^\textrm{b}(X)$. Since  is a free group, we have the vanishing $H^3(G, \mathbb {C}^*)=0$, and hence the assertion holds. $\square$

### Remark 5.4

Although [7, Theorem 2.1] is stated only for finite groups, its proof works for infinite groups without any modifications.

### Lemma 5.5

([7, Lemma 2.2]) Let ,  be functors, and let $\alpha :f \rightarrow f'$, $\beta :g \rightarrow g'$ be natural transforms. Then the following diagram commutes:

### Proposition 5.6

There is an $\{x^\pm \}$-coordinatized local system ${\mathfrak L}$ on  with assignments

### Proof

Let ${\mathfrak K}$ be the local system of categories constructed in Lemma 5.3. By definition, it consists of the datafor all elements . Moreover, we have the following commutative diagrams:5.3for all .

We extend this local system ${\mathfrak K}$ to an $\{x^\pm \}$-coordinatized local system ${\mathfrak L}$. First observe that any morphism *g* in the groupoid  can be uniquely written as follows:5.4To the morphism (5.4), we associate the functorFor each pair *g*, *h* of composable morphisms, we associate a natural transform $\theta _{g, h} :\rho _g\rho _h \rightarrow \rho _{gh}$ as follows:

- When $g=\gamma$, $h=\gamma ^{-1}$, then we define $\theta _{\gamma , \gamma ^{-1}}$ to be the adjoint counit
- When we have $g=\gamma ^{\epsilon }g'\gamma ^{-1}$, $h=\gamma h'\gamma ^\delta$ for some $\epsilon , -\delta \in \{0, 1\}$, , then we define $\theta _{g, h}$ as  where  is the adjoint counit.
- Otherwise, we put .

We need to check the commutativity of the diagram (3.1) for all composable paths *g*, *h*, *k*. We prove it only for the case when we have $h=\gamma h'$ with . The other cases can be proved in a similar way. As in (5.4), we writeConsider the following diagram:The upper and the lower triangles are commutative by the definitions of $\theta _{g, h}$ and $\theta _{g, hk}$; the left square is commutative by Lemma 5.5; the right square is commutative by the commutativity of (5.3). Hence we conclude that the whole diagram commutes as required. $\square$

Next we shall extend the local system ${\mathfrak L}$ of categories to a perverse schober on $\mathbb {P}^1$. Let us take a smooth hyperplane section , and denote by $i :C \hookrightarrow X$ the natural inclusion. We also take a general linear section $j :\mathbb {C}^{n+1} \hookrightarrow \mathbb {C}^{n+2}$. Note that by using the equivalences (see (4.4))we have the natural push-forward functor $j_* :\textrm{HMF}^{\,\mathrm gr}(W|_{\mathbb {C}^{n+1}}) \rightarrow \textrm{HMF}^{\,\mathrm gr}(W)$.

### Theorem 5.7

There exists a perverse schober ${\mathfrak P}$ on  with the following properties:

1. The restriction of the schober ${\mathfrak P}$ to  coincides with the local system ${\mathfrak L}$ of categories constructed in Proposition 5.6.
2. Restricting the schober ${\mathfrak P}$ to small disks around $\infty , 1, 0$, we obtain the spherical functors and the signs 5.5 respectively.

### Remark 5.8

Our choices of spherical functors (5.5) are compatible with the general result in Theorem 2.12. Indeed, by [29], we have an SOD

$$\begin{aligned} \textrm{HMF}^{\,\mathrm gr}(W|_{\mathbb {C}^{n+1}})=\langle D^\textrm{b}(C), D^\textrm{b}({{\,\textrm{pt}\,}}) \rangle , \end{aligned}$$

and the dual twist of the spherical functor  is exactly the composition .

Our point is that we have explicit descriptions of the source categories of spherical functors in terms of derived categories and matrix factorization categories, rather than abstract dg categories.

**Fig. 2:**
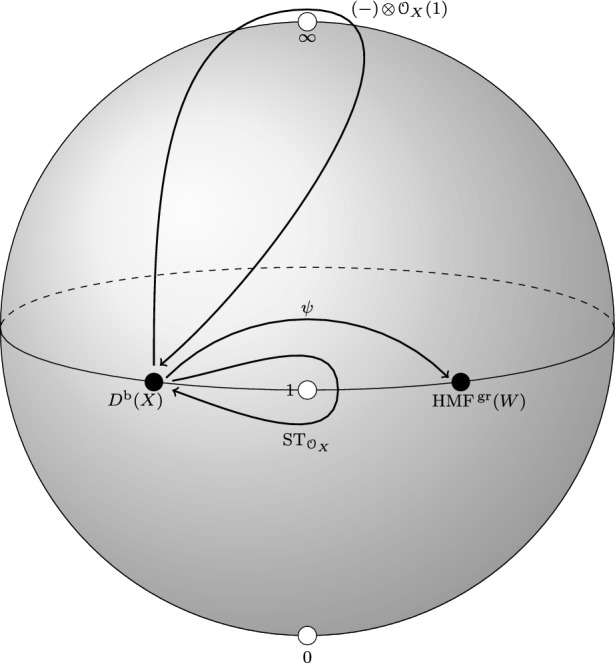
Local system of categories

### Proof of Theorem 5.7

We apply Proposition 3.10 to the local system ${\mathfrak L}$ three times. It is enough to show that the functors (5.5) are spherical and their twists coincide with the autoequivalencesrespectively (cf. Remark 3.5 and Example 3.9).

We only prove the assertion for the functor5.6namely, we show that it lifts to a spherical dg functor and the corresponding twist autoequivalence of $D^\textrm{b}(X)$ is isomorphic to . We take dg enhancements of the categories $\textrm{HMF}^{\,\mathrm gr}(W)$ and $\textrm{HMF}^{\,\mathrm gr}(W|_{\mathbb {C}^{n+1}})$ to be  and , respectively. Note that they also give Morita enhancements, since the categories $D^\textrm{b}(X)$ and $\textrm{HMF}^{\,\mathrm gr}(W|_{\mathbb {C}^{n+1}})$ are idempotent complete. Since $j :\mathbb {C}^{n+1} \hookrightarrow \mathbb {C}^{n+2}$ is a closed embedding, we have the functorwhich gives a dg enhancement of the functor (5.6).

Next we construct dg lifts of the left and right adjoint functorsFor this we take dg enhancements of $\textrm{HMF}^{\,\mathrm gr}(W)$ and $\textrm{HMF}^{\,\mathrm gr}(W|_{\mathbb {C}^{n+1}})$ as  and , respectively. Then we have the dg enhancementSimilarly, we have the enhancement of the functor , where  is the $\mathbb {C}^*$-equivariant line bundle of weight 1. Note that the dg enhancements $D^{\textrm{abs}}{{\,\textrm{fact}\,}}(-)$ and ${{\,\textrm{vect}\,}}(-)$ are quasi-equivalent (see Proposition 4.10).

Let us consider the twist *T* of $j_*$, which fits into the exact triangle5.7of quasi-functors on . Applying the underlying exact functors of (5.7) to an object $E \in \textrm{HMF}^{\,\mathrm gr}(W)$, we obtain a triangle

5.8 $$\begin{aligned} E(\chi _1)|_{\mathbb {C}^{n+1}}[-1] \rightarrow E \rightarrow T(E) \end{aligned}$$

in $\textrm{HMF}^{\,\mathrm gr}(W)$. The exact triangle (5.8) is isomorphic to the triangle obtained by tensoring *E* to the sequence . In other words, we have isomorphisms5.9between functors. In particular, the functor [*T*] is an autoequivalence of $\textrm{HMF}^{\,\mathrm gr}(W)$. Similarly, we can check that the endofunctor [*F*] of $\textrm{HMF}^{\,\mathrm gr}(W|_{\mathbb {C}^{n+1}})$ is an equivalence, where *F* is the cotwist of $j_*$ defined by the triangleIndeed, we can show that

5.10 $$\begin{aligned} {[}F] \simeq S_{\textrm{HMF}^{\,\mathrm gr}(W|_{\mathbb {C}^{n+1}})}[-n-1], \end{aligned}$$

where $S_{(-)}$ denotes the Serre functor.

The above arguments show that the push-forward functor $j_*$ is a spherical functor. Moreover, its twist is isomorphic to the degree shift functor by (5.9). Since $\psi ^{-1} :\textrm{HMF}^{\,\mathrm gr}(W) \rightarrow D^\textrm{b}(X)$ is an equivalence, the composition  is also a spherical functor. By applying  to the triangle (5.7), we obtainRecall from Theorem 5.1 that the degree shift functor $\tau$ corresponds to the autoequivalence  via the equivalence $\psi$. It follows that the twist of the spherical functor  isas required. $\square$

### Proposition 5.9

The schober ${\mathfrak P}$ constructed in Theorem 5.7 is *n*-Calabi–Yau.

### Proof

The local system ${\mathfrak L}$ clearly satisfies the Calabi–Yau property.

By the isomorphism (5.10), the schober ${\mathfrak P}$ restricted to a disk around 0 is *n*-Calabi–Yau. Similarly, one can check that it is *n*-Calabi–Yau around the points $\infty , 1$. $\square$

## Decategorification

In this section, we prove that the decategorifications of our perverse schobers coincide with the intersection complexes.

### Intersection complex

We first recall the description of intersection complexes under the equivalence in Proposition 3.3:

#### Proposition 6.1

([12, Proposition 3.2]) Given a local system *L* on  with fiber *F* and monodromy *m*, the intersection complex $\textrm{IC}(L) \in \textrm{Perv}(\Delta , 0)$ corresponds to the diagram6.1where  denotes the *m*-invariant part, and $u :F \rightarrow F/F^m$ denotes the quotient map.

#### Remark 6.2

In [12], Donovan uses a slightly different version of the quiver description of the category $\textrm{Perv}(\Delta , 0)$. Namely, our category  is the category of data $(D, D_1, u, v)$ such that  is an isomorphism. On the other hand, Donovan [12, Proposition 3.1] considers the condition that  is an isomorphism. As a result, the map $F/F^m \rightarrow F$ in (6.1) is ${{{\,\textrm{id}\,}}}-m$ in our case, while it is $m-{{\,\textrm{id}\,}}$ in [12, Proposition 3.2].

The following lemma is useful for our purpose:

#### Lemma 6.3

Let *X* be a smooth projective variety, $U \subset X$ be an open subset, and *L* be a local system on *U*.

Given a perverse sheaf *P* on *X*, the following conditions are equivalent:

1. We have an isomorphism $P \simeq \textrm{IC}(L)$.
2. There exists an analytic open covering $X=\bigcup _i V_i$ such that for every *i*, we have an isomorphism .

#### Proof

This is a well-known property, and follows from the characterization of the intersection complexes given in [28, Definition 8.4.3 (2)], together with the fact that the category of perverse sheaves forms a stack in analytic topology, see e.g., [28, Remark 8.2.11]. $\square$

### Decategorification of perverse schobers

In this subsection, we consider the decategorification of our schober when $n \geqslant 2$. Let  be the hyperplane class. We also denote by *H* its restrictions to *X* and *C*.

#### Definition 6.4

1. We define the *H-part* $\Lambda _H(X)$ (resp. $\Lambda _H(C)$) of the cohomology $H^*(X, \mathbb {Q})$ (resp. $H^*(C, \mathbb {Q})$) to be the subring generated by the hyperplane class *H*.
2. We define the *H-part* $\Lambda _H(W|_{\mathbb {C}^{n+1}})$ of the numerical Grothendieck group $K_{{{\,\textrm{num}\,}}}(\textrm{HMF}^{\,\mathrm gr}(W|_{\mathbb {C}^{n+1}}) )_\mathbb {Q}$ to be .

#### Remark 6.5

By [29], we have an SOD

$$\begin{aligned} \textrm{HMF}^{\,\mathrm gr}(W|_{\mathbb {C}^{n+1}})=\langle D^\textrm{b}(C), D^\textrm{b}({{\,\textrm{pt}\,}}) \rangle . \end{aligned}$$

Hence we have a natural embedding

$$\begin{aligned} \Lambda _H(W|_{\mathbb {C}^{n+1}}) \hookrightarrow K_{{{\,\textrm{num}\,}}}(\textrm{HMF}^{\,\mathrm gr}(W|_{\mathbb {C}^{n+1}}) )_\mathbb {Q}. \end{aligned}$$

The proof of the following lemma is straightforward:

#### Definition-Lemma 6.6

The following assertions hold:

1. The local system ${\mathfrak L}$ of categories in Proposition 5.6 induces a local system on  whose fiber is $\Lambda _H(X)$. We denote it by *L*.
2. The spherical functors with the signs in (5.5) induce linear maps
  6.2 $$\begin{aligned} \begin{aligned}&i_* :\Lambda _H(C) \rightarrow \Lambda _H(X), \quad i^! :\Lambda _H(X) \rightarrow \Lambda _H(C), \\&\mathbb {Q}\rightarrow \Lambda _H(X), \quad \Lambda _H(X) \rightarrow \mathbb {Q}, \\&j_* :\Lambda _H(W|_{\mathbb {C}^{n+1}}) \rightarrow \Lambda _H(X), \quad j^* :\Lambda _H(X) \rightarrow \Lambda _H(W|_{\mathbb {C}^{n+1}}), \end{aligned} \end{aligned}$$
  which define perverse sheaves on $(\Delta , 0)$.
3. The local system *L* in (1) and the data (6.2) define a perverse sheaf *P* on $\mathbb {P}^1$.

We call *L*, *P* the *decategorifications* of ${\mathfrak L}, {\mathfrak P}$, respectively.

#### Theorem 6.7

Suppose that $n \geqslant 2$. Let *L*, *P* be the decategorifications of ${\mathfrak L}, {\mathfrak P}$ as in Definition-Lemma 6.6.

Then we have an isomorphism $P \simeq \textrm{IC}(L) \in \textrm{Perv}(\mathbb {P}^1)$. Moreover, the perverse sheaf *P* is Verdier self-dual.

#### Proof

By Lemma 6.3, it is enough to check the isomorphism of the perverse sheaves $\textrm{IC}(L)$ and *P* in the neighborhoods of the points $0, 1, \infty$. To show this, we apply Proposition 6.1 to the data in (6.2).

We only prove the assertion for6.3namely, we prove that this is isomorphic to the diagram (6.1) with  and . We claim that $F^m=0$ in this case, and hence the diagram (6.1) becomes6.4To prove the claim, first note that $F^m=0$ if and only if $F^{(m^{-1})}=0$. Recall that we have the triangleHence we have

$$\begin{aligned} m^{-1}(\textbf{x}) =\textbf{x}.e^H-\chi (\textbf{x}.e^H)(1, 0, \dots , 0) \end{aligned}$$

for any $\textbf{x}=(x_0, \dots , x_n) \in \Lambda _H(X)$. Assume that $\textbf{x}$ is fixed by $m^{-1}$. Firstly, the second to the last components of $\textbf{x}$ and $m^{-1}(\textbf{x})$ must be equal, which is only possible when $x_0=x_1= \cdots =x_{n-1}=0$. Then the first component of $m^{-1}(\textbf{x})$ is $-\chi (\textbf{x}.e^H)=-x_n$, while we have seen $x_0=0$. Hence the *m*-fixed part is trivial as claimed.

Next observe that we have the following morphism in the category  (cf. Definition 3.2):Hence, to prove that the diagrams (6.3) and (6.4) are isomorphic, it is enough to show that the map  is an isomorphism. Since we assume that $n \geqslant 2$, $C \subset X$ is a connected smooth variety, and the vector spaces  and $\Lambda _H(X)$ have the same dimension. Hence it is enough to show that $j_* j^*={{{\,\textrm{id}\,}}}-m$ is an isomorphism. Since we have provedthe map $j_*j^*$ is an injective endomorphism, hence it is an isomorphism as required.

The Verdier self-duality of *P* follows from Proposition 5.9, see also Remark 3.13. $\square$

#### Remark 6.8

When $n=1$, *X* is an elliptic curve and *C* consists of three points. Then we would have $\dim \Lambda _H(W|_{\mathbb {C}^2})=4 > 2=\dim \Lambda _H(X)$. Hence the above computation shows that *P* and $\textrm{IC}(L)$ are not isomorphic. This is related to the fact that  has degree three, and hence does not generate the Picard group when $n=1$.

We will modify the construction in the next section to get the categorification of the intersection complex for elliptic curves.

#### Remark 6.9

Even when $n \geqslant 2$, if we consider the full numerical *K*-groups instead of the *H*-parts, the result fails in general. Indeed, it may happen that

$$\begin{aligned} \dim K_{{{\,\textrm{num}\,}}}(\textrm{HMF}^{\,\mathrm gr}(W|_{\mathbb {C}^{n+1}}))_\mathbb {Q}> \dim K_{{{\,\textrm{num}\,}}}(X)_\mathbb {Q}. \end{aligned}$$

## Mirror symmetry for elliptic curves

In this section, we denote by *X* a smooth elliptic curve. We construct perverse schobers in both *A*- and *B*-models, which are identified via the homological mirror symmetry for an elliptic curve.

A key observation is that, in the case of elliptic curves, our schober only consists of spherical objects, while in higher dimension we have more general spherical functors.

### Perverse schober on the *B*-side

The following is a straightforward modification of the construction in Theorem 5.7:

#### Proposition 7.1

Fix a point $p \in X$. There exists a perverse schober ${\mathfrak P}^B$ on  satisfying the following properties:

1. The schober ${\mathfrak P}^B$ restricted to the open subset  is isomorphic to a local system ${\mathfrak L}^B$ of categories with the fiber $D^\textrm{b}(X)$, defined by the assignments
2. Restricting the schober ${\mathfrak P}^B$ to small disks around $\infty , 1, 0$, we obtain the spherical functors with the signs  respectively.
3. By taking the Grothendieck groups, we obtain a local system $L^B$ and a perverse sheave $P^B$ in the usual sense. We have $P^B \simeq \textrm{IC}(L^B)$.

#### Proof

The proof is almost identical to that of Theorem 5.7. The first difference is that we use  instead of the degree three line bundle . The second difference is that the skyscraper sheaf  is spherical for an elliptic curve *X*, whose twist is isomorphic to .

Finally, to construct a spherical functor $\langle D^\textrm{b}({{\,\textrm{pt}\,}}), D^\textrm{b}({{\,\textrm{pt}\,}}) \rangle \rightarrow D^\textrm{b}(X)$ whose dual twist is , we use Theorem 2.12. $\square$

### Perverse schober on the *A*-side

Here, we construct the *mirror* perverse schober using the homological mirror symmetry for an elliptic curve.

Let  be an elliptic curve, where $\tau$ is an element of the upper half plane. Then its *mirror* is defined to be a pair , where $T=\mathbb {C}/\mathbb {Z}^{\oplus 2}$ is a torus, and  is a *complexified Kähler form*. We denote by $\pi :\mathbb {C}\rightarrow T$ the quotient map.

We briefly recall the definition of the Fukaya category $D^\pi \textrm{Fuk}(X^\vee )$, following [30]. An object of the Fukaya category $D^\pi \textrm{Fuk}(X^\vee )$ is isomorphic to a tuple $(L, \alpha , M)$, where

- $L \subset T$ is a special Lagrangian submanifold,
- $\alpha \in \mathbb {R}$ is a real number such that the equation
  $$\begin{aligned} L=\pi \bigl (\{ z(t) \in \mathbb {C}: z(t)=z_0+e^{i\pi \alpha }t, \, t \in \mathbb {R}\}\bigr ) \end{aligned}$$
  holds for some $z_0 \in \mathbb {C}$.
- *M* is a $\mathbb {C}$-local system on *L* whose monodromy has eigenvalues in the unit circle.

Morphisms in the Fukaya category are defined to be the morphisms between local systems restricted to the intersection of Lagrangian submanifolds. Finally, the $A_\infty$-structure is defined by using the complexified Kähler form $\omega _\mathbb {C}$ and the moduli spaces of pseudo-holomorphic disks in $X^\vee$.

We denote by  (resp. , where $L_0$ (resp. $L_1$) is the image of the imaginary axis (resp. the real axis) in $\mathbb {C}$ under the projection map $\pi :\mathbb {C}\rightarrow T$.

#### Theorem 7.2

([30]) There exists an equivalence

7.1 $$\begin{aligned} \Phi _{{{\,\textrm{mirror}\,}}} :D^\textrm{b}(X) \xrightarrow { \ \sim \ } D^\pi \textrm{Fuk}(X^\vee ), \end{aligned}$$

which sends  to , respectively, where $e \in X$ is the origin.

In particular, the objects , $i=0, 1$, are spherical objects. We denote by  the corresponding spherical twists, which are called the *Dehn twists along* .

Combining Proposition 7.1 and Theorem 7.2, we obtain the following:

#### Theorem 7.3

There exists a perverse schober ${\mathfrak P}^A$ on  satisfying the following properties:

1. The schober ${\mathfrak P}^B$ restricted to the open subset  is isomorphic to a local system ${\mathfrak L}^A$ of categories with the fiber $D^\pi \textrm{Fuk}(X^\vee )$, defined by the assignments
2. Restricting the schober ${\mathfrak P}^A$ to small disks around $\infty , 1, 0$, we obtain the spherical functors with the signs  respectively.

Under the mirror symmetry equivalence (7.1), the perverse schober ${\mathfrak P}^A$ is identified with the schober ${\mathfrak P}^B$ with $p=e \in X$.

## Orlov equivalences via VGIT

In this section, we review the proof of Orlov’s theorem (Theorem 5.1) via the magic window theorem and Knörror periodicity, following [5, Section 7]. This approach naturally gives an example of spherical pairs in the sense of Definition 3.14.

### VGIT and window shift autoequivalences

Let us put , , and define an action of *G* on *X* as follows:Let $\chi ^{\pm } :G \rightarrow \mathbb {C}^*$ be characters defined as  By taking the GIT quotients with respect to the characters $\chi ^\pm$, we obtainwhere $\mathbb {C}^*$ acts on the fiber of  by weight one, and $\mathbb {C}^*$ acts on $\mathbb {C}^{n+2}$ by weight one. We have natural embeddings $Y^\pm \hookrightarrow [Y/G]$.

We further take a general homogeneous polynomial $W \in \mathbb {C}[x_1, \cdots , x_{n+2}]$ of degree $n+2$, and define a function $Q_W :Y \rightarrow \mathbb {C}$ by . Then $Q_W$ is a $\eta$-semi-invariant function, where $\eta :G \rightarrow \mathbb {C}^*$ is a character defined as $\eta (\lambda , \mu )=\mu$. Indeed, we haveHence the data $(Y, \eta , Q_W)^G$ defines a gauged Landau–Ginzburg model.

#### Definition 8.1

1. For an interval *I*, we define the *window subcategory*
  to be a triangulated subcategory generated by factorizations (4.1) with terms  as $\mathbb {C}^*$-equivariant sheaves on *Y*, where $\mathbb {C}^*$ acts on *Y* via the inclusion  into the first factor.
2. For an integer $w \in \mathbb {Z}$, we put

The following is a special case of the main theorem of [5, 18]:

#### Theorem 8.2

([5, 18]) For each integer $w \in \mathbb {Z}$, the compositionsare equivalences, where ${{\,\textrm{res}\,}}^\pm$ denote the restriction functors to the semi-stable loci $Y^\pm$. In particular, we have an equivalence

#### Definition 8.3

For each integer $w \in \mathbb {Z}$, we putand call it as the *window shift autoequivalence*.

In the following, we will interpret the window shift autoequivalences as autoequivalences of $D^\textrm{b}(X)$ under the Knörror periodicity, where  is a smooth Calabi–Yau hypersurface of dimension *n*. We have the following diagram:where *p*, *q* denote the projections, $\gamma , \gamma _X$ are the inclusions as zero sections, and $i, i_X$ are the natural inclusions.

We use the following result:

#### Proposition 8.4

([19, Proposition 3.4]) The window shift autoequivalence $\Phi _{w}$ fits into the following exact triangle:8.1In other words, $\Phi _{w}$ is the spherical twist around .

Recall from Theorem 4.16 that we have an equivalence8.2

#### Proposition 8.5

Let $w \in \mathbb {Z}$ be an integer. Under the Körror periodicity equivalence (8.2), the window shift autoequivalence $\Phi _w$ corresponds to the spherical twist  on $D^\textrm{b}(X)$.

#### Proof

To simplify the notation, we put . Since we have an isomorphismof functors (see e.g., [19, Lemma 3.2]) for each $k \in \mathbb {Z}$, we may assume $w=-n-1$.

We prove a functorial isomorphism  for each object $E \in D^\textrm{b}(X)$. By applying the functor $i_*p^*$ to the defining trianglewe obtain8.3On the other hand, by the triangle (8.1), we have the exact triangle8.4We compute the complex  as follows:8.5where the first isomorphism follows from the adjunction; the second isomorphism follows from Lemma 8.6 (2) below; the third isomorphism follows from the adjunction and the fact that ; the last isomorphism follows from .

Moreover, we have an isomorphism8.6by Lemma 8.6 (1) below.

By the isomorphisms (8.5) and (8.6), it follows that the triangles (8.3) and (8.4) are isomorphic, as required. $\square$

We have used the following lemma in the above proof:

#### Lemma 8.6

Put , . The following statements hold:

1. We have an isomorphism
2. We have an isomorphism

#### Proof

Put , and let  be the tautological section. By Lemma 4.15, we havewhich proves the first assertion.

Similarly, we havewhere the second equality follows from the vanishing , hence the second assertion holds. $\square$

We can now give the proof of Orlov’s theorem:

#### Proof of Theorem 5.1

As before, we put . The existence of the equivalence

$$\begin{aligned} D^\textrm{b}(X) \simeq \textrm{HMF}^{\,\mathrm gr}(W) \end{aligned}$$

follows from Theorem 8.2 together with the equivalences (8.2) and (4.4).

For the second statement, consider the following commutative diagram:8.7where  denotes the $\mathbb {C}^*$-equivariant line bundle of weight one, with respect to the $\mathbb {C}^*$-action via the inclusion  to the first factor.

By (4.4), the autoequivalencecorresponds to the degree shift equivalence $\tau$ under the natural equivalenceOn the other hand, the autoequivalence  on $D^\textrm{abs}[{{\,\textrm{fact}\,}}_{\,\mathbb {C}^*}(V, \chi _1, Q_W)]$ corresponds to the autoequivalence  on $D^\textrm{b}(X)$ via the Knörror periodicity (8.2).

By the above observations, the commutativity of the diagram (8.7) implies thatwhere the last equality follows from Proposition 8.5. $\square$

### Spherical pairs from VGIT

We end this section by constructing an example of spherical pairs using the theory of [5, 18], following [12]. Letbe the unstable loci with respect to the characters $\chi ^+, \chi ^-$, respectively. Let $j^\pm :S^\pm \hookrightarrow Y$ denote the inclusions.

#### Proposition 8.7

([12, 19]) We have a spherical pair8.8such that the induced autoequivalence on $D^\textrm{b}(X)$ is isomorphic to .

#### Proof

By [19, Equation (3)], applied to the two different KN stratificationswe have a pair of semi-orthogonal decompositions:8.9The proofs of [12, Theorem 4.4, Proposition 4.5] show that the pair (8.9) defines a spherical pair. Moreover, by Theorem 8.2 and the equivalences (8.2) and (4.4), we haveHence we obtain the spherical pair as in (8.8).

It remains to compute the induced autoequivalence on $D^\textrm{b}(X)$. By construction, the induced autoequivalence on  is the spherical twist around the object . By Propositions 8.4 and 8.5, it corresponds to the spherical twist  under the Knörror periodicity equivalence (8.2). $\square$

#### Corollary 8.8

The perverse schober in Theorem 5.7 upgrades to the spherical pair (8.8) around the point $0 \in \mathbb {P}^1$.
